# Staufen2 dysregulation in neurodegenerative disease

**DOI:** 10.1016/j.jbc.2025.108316

**Published:** 2025-02-13

**Authors:** Sharan Paul, Warunee Dansithong, Karla P. Figueroa, Mandi Gandelman, Pravin Hivare, Daniel R. Scoles, Stefan M. Pulst

**Affiliations:** Department of Neurology, University of Utah, Salt Lake City, Utah, USA

**Keywords:** RNA-binding protein, Staufen, autophagy, neurodegeneration, neurodegenerative diseases and ataxia

## Abstract

Staufen2 (STAU2) is an RNA-binding protein that controls mRNA trafficking and expression. Previously, we showed that its paralog, Staufen1 (STAU1), was overabundant in cellular and mouse models of neurodegenerative diseases and amyotrophic lateral sclerosis (ALS) patient spinal cord. Here, we investigated features of STAU2 that might parallel STAU1. STAU2 protein, but not mRNA, was overabundant in spinocerebellar ataxia type 2 (SCA2), ALS/frontotemporal dementia patient fibroblasts, ALS patient spinal cord tissues, and in central nervous system tissues from SCA2 and ALS animal models. Exogenous expression of STAU2 in human embryonic kidney 293 cells activated mechanistic target of rapamycin (mTOR) and stress granule formation. Targeting *STAU2* by RNAi normalized mTOR in SCA2 and C9ORF72 cellular models. The microRNA miR-217, previously identified as downregulated in SCA2 mice, targets the *STAU2* 3′-UTR. We now demonstrate that exogenous expression of miR-217 significantly reduced STAU2 and mTOR levels in cellular models of neurodegenerative disease. These results suggest a functional link between STAU2 and mTOR signaling and identify a major role for miR-217 that could be exploited in therapeutic development.

RNA-binding proteins (RBPs) have multiple functions in the cell ranging from splicing to regulating mRNA translation and degradation to RNA transport. RNA recognition motifs in RBPs utilize aggregate-promoting low-complexity domains to form various ribonucleoprotein (RNP) granules, among which include stress granules (SGs), transport granules, and P-bodies. Their unique structure predisposes to the formation of protein–RNA aggregates and provides insights into protein misfolding in neurodegenerative diseases (NDDs). Mutations in several RBPs cause RNP granules and many Mendelian forms of NDDs (1, 2).

Direct interaction studies of RBPs can provide insight into functionality of protein partners and the development of potential therapeutic targets. Ataxin 2 (ATXN2), an RBP that when mutated by polyglutamine (polyQ) expansion causes spinocerebellar ataxia type 2 (SCA2) (3, 4, 5, 6), was shown to bind to the amyotrophic lateral sclerosis (ALS)–linked Tar DNA-binding protein 43 (TDP-43) (7). This led us to search for other interactors and potential therapeutic targets by purifying the ATXN2 protein complex using immunoprecipitation and mass spectrometry. In addition to RNA-binding fox-1 homolog 1 (RBFOX1/A2BP1) (8), one of the most intriguing ATXN2 interactors was Staufen1 (STAU1), a double-stranded RBP and SG component (9). STAU1 functions in mRNA transport in neuronal dendrites, and other cells in vertebrates (10, 11, 12, 13, 14).

Previously, we showed that STAU1 was overabundant in cells expressing mutant polyQ-expanded ATXN2 and in other *in vitro* and *in vivo* models of NDDs as well as in human ALS spinal cord (9, 15, 16, 17). STAU1 overabundance was attributed to reduced degradation *via* autophagy and was not because of increased transcription. We established a link between STAU1 and autophagy regulation by showing enhanced translation of *mTOR* (mechanistic target of rapamycin) mRNA (a master regulator of autophagy) through 5′UTR interaction (16). Reducing STAU1 levels attenuated *in vitro* cellular phenotypes, restored SCA2 mouse cerebellar molecular phenotypes, and improved their motor behavior, and normalized the mTOR activity of the unfolded protein response in SCA2 and ALS-TDP-43 mice (9, 15, 16, 17). These studies suggest that targeting STAU1 may be an effective approach to treating NDDs.

Although the fly has only one staufen gene, a second gene arose in evolution through duplication. Two Staufen paralogs, STAU1 and STAU2, both function as double-stranded RBPs and are encoded by distinct genes. Both proteins share an overall 50% amino acid sequence identity (18, 19). STAU2 possesses four double-stranded mRNA-binding domains (dsRBDs), followed by a tubulin-binding domain and a C-terminal and noncanonical dsRBD-like domain, in contrast to only three dsRBDs in STAU1 (18). Even though dsRBDs 3 and 4 are highly similar (amino acid sequence) between STAU1 and STAU2 (78% and 81%, respectively) (20, 21, 22, 23), both proteins exhibit distinct and partially overlapping (∼30%) sets of target mRNAs, as observed in a human embryonic kidney 293T (HEK293T) cell culture model using a genome-wide approach, indicating distinct and complementary functions (19). These overlapping mRNA populations encode proteins involved in multiple pathways, including cell metabolism, cellular physiological processes, localization and transport, transcription, and alternative splicing (19). The greater prevalence of distinct mRNAs either for STAU1 or STAU2 supports the idea that both Staufen proteins have conserved convergent functions, as they target different populations of RNA granules in mature neurons (22, 24). For instance, STAU1, but not STAU2, functions in the late phase of forskolin-induced long-term potentiation (14, 25, 26), whereas STAU2, but not STAU1, functions in metabotropic glutamate receptor–mediated long-term depression (25). STAU1 exhibits expression across many cell types, including neurons, whereas STAU2 is enriched in heart and brain cells, including Purkinje cells (9, 20, 21, 22, 27).

However, STAU1 and STAU2 also share biological functions, including the microtubule-dependent transport of RNAs (11, 12) and the formation and maintenance of dendritic spines of hippocampal neurons (14, 25, 26, 28, 29). Both STAU1 and STAU2 are recruited to cytoplasmic inclusions in neurons with C9ORF72 mutations and brain oligodendrocytes and modulate SG dynamics (22, 24, 30, 31). Similar to STAU1, STAU2 interacts with ATP-dependent RNA helicase up-frameshift 1 (UPF1) and regulates the stability of specific transcripts through interaction with their 3′UTRs, a mechanism referred to as Staufen-mediated mRNA decay (32). A recent transcriptome-wide splicing analysis demonstrated significant alternative splicing events in ALS-relevant TDP-43-induced pluripotent stem cell–derived motor neuron models. Several RBPs, including STAU2, exhibited differential splicing in ALS *versus* control TDP-43-induced pluripotent stem cell–derived motor neurons (33).

Our interest in STAU2 was also sparked by prior studies examining mRNA and miRNA expression in SCA2 mouse models (34, 35, 36, 37). One of the highly dysregulated miRNAs was miR-217, which is predicted to potentially target STAU2 among other mRNAs. The role of STAU2 in autophagy and neurodegeneration, however, has remained poorly characterized.

In this study, we show that STAU2 steady-state levels are increased in SCA2 and ALS/frontotemporal dementia (FTD)-C9ORF72 cellular and mouse models as well as in spinal cord tissues from patients with ALS. We establish a role for STAU2 in regulating mTOR signaling, which was normalized by *STAU2* silencing. In addition, we analyzed the interaction of miR-217 abundance with *STAU2* mRNA and established a novel role for miR-217 in regulating STAU2 expression. Our results describe STAU2 overabundance as a common feature across multiple NDDs.

## Results and discussion

### STAU2 levels are increased in NDDs

**Figure 1:**
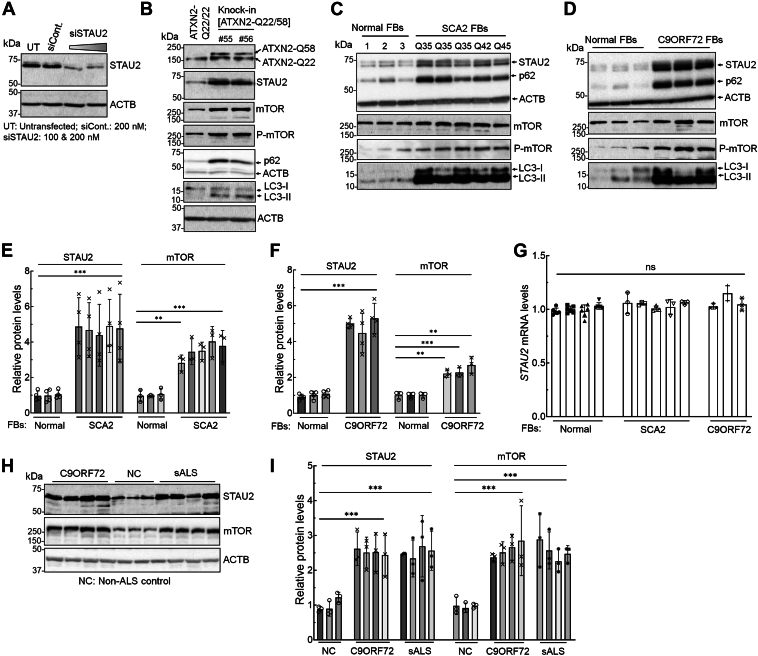
**Increased STAU2 levels and abnormal mTOR signaling in NDD patient cell lines.***A*, characterization of the STAU2 antibody. HEK293 cells were transfected with an STAU2 siRNA or a scrambled control siRNA (siCont) and analyzed 4 days post-transfection by Western blotting. STAU2 bands of 62 and ∼64 kDa were observed, which were reduced by the STAU2 siRNA but not siCont, indicating antibody specificity. *B*–*F*, Western blot analysis of protein extracts from CRISPR–Cas9 edited HEK293-ATXN2-Q58 KI cells (*B*), FBs from individuals with SCA2 mutations (three with Q35, Q42, and Q45 repeats) (*C*), and ALS–FTD patients with *C9ORF72* expansions (*D*). All show increased levels of STAU2, mTOR, P-mTOR, p62, and LC3-II compared with controls. Quantified STAU2 and mTOR average fold changes are shown in *E* and *F*. *G*, *STAU2* mRNA levels are unaltered in-patient cell lines. qRT–PCR analyses of RNA extracts from the SCA2 and C9ORF72 FB cells (*C* and *D*). *H* and *I*, spinal cord tissues from patients with *C9ORF72* expansions and sALS showing increased STAU2 and mTOR levels compared with non-ALS controls (NC) on Western blots (*H*). Quantification of STAU2 and mTOR levels on Western blots (*I*). Each lane represents an individual patient’s cell line or tissue (*C*, *D*, and *H*). ACTB was used as a loading control, and the representative blots of three independent experiments are shown. Two-way ANOVA followed by Bonferroni tests for multiple comparisons. Data are mean ± SD, ns, *p* > 0.05; *∗∗∗p* < 0.001. ALS, amyotrophic lateral sclerosis; FB, fibroblast; FTD, frontotemporal dementia; HEK293, human embryonic kidney 293 cell line; KI, knock-in; mTOR, mechanistic target of rapamycin; NDD, neurodegenerative disease; ns, not significant; qRT–PCR, quantitative RT–PCR; sALS, sporadic amyotrophic lateral sclerosis; STAU2, Staufen2.

As STAU1 had shown increased protein abundance (9, 15, 16), we tested STAU2 steady-state protein levels in cell lines from individuals with inherited NDDs, including SCA2 and ALS–FTD (Fig. 1). We first assessed the specificity of the STAU2 antibody by Western blotting of protein extracts from HEK293 cells that were treated for 4 days with a *STAU2* siRNA *versus* a scrambled control siRNA (siCont). The predominant STAU2 band at 62 kDa was strongly detected with a lighter intensity band appearing at ∼64 kDa, and these were unmodified by the scrambled control siRNA but were strongly reduced in cells treated with the *STAU2* siRNA (Fig. 1*A*). To measure STAU2 levels, we used a previously described HEK293-ATXN2-Q22/Q58 (ATXN2-Q58) knock-in (KI) cell line as well as fibroblast (FB) lines derived from SCA2 patients with ATXN2 repeat expansions (Q35, Q42, and Q45 polyQ repeats) (9, 15, 16). In all cell lines, STAU2 levels were increased (Fig. 1, *B* and *C*). We also observed that total and active phospho-mTOR (P-mTOR) levels were increased, as were levels of SQSTM1/p62 and LC3-II, consistent with our previous observations of impaired autophagy in SCA2 cellular and mouse models (Fig. 1, *B* and *C*) (9, 15, 16).

As STAU1 levels were increased in ALS–FTD patients with *C9ORF72* repeat expansions (15, 16), we examined STAU2 levels in these cell lines. In all cases, STAU2 levels were significantly increased as were mTOR, P-mTOR, p62, and LC3-II (Fig. 1*D*). Quantification of average fold changes for STAU2 and mTOR in FBs from SCA2 and ALS–FTD patients is shown in Figure 1, *E* and *F*. *STAU2* mRNA levels were unchanged in both FBs from SCA2 and ALS–FTD patients indicating that the overabundance of STAU2 protein is post-translational (Fig. 1*G*).

We also examined STAU2 levels in human patient tissues. We obtained spinal cord tissues from patients with *C9ORF72* expansions and from patients with sporadic ALS (sALS) and determined STAU2 levels by Western blotting. Compared with spinal cord tissue from non-ALS controls, all ALS samples showed increased STAU2 and mTOR levels (Fig. 1*H*). Quantification of STAU2 and mTOR changes is shown in Figure 1*I*. The elevation of STAU2 in both C9ORF72-associated ALS and sALS likely results from disruptions in protein stability and RNA metabolism, a common pathological mechanism in ALS. In C9ORF72-associated ALS–FTD, RNA foci formation, altered splicing, and SG dynamics contribute to RNA dysregulation (38). Similar mechanisms may occur in sALS because of stress responses and RBP aggregation, leading to increased STAU2 abundance as part of a compensatory or maladaptive response to maintain RNA homeostasis.

### STAU2 is overabundant in mouse models of neurodegeneration

**Figure 2:**
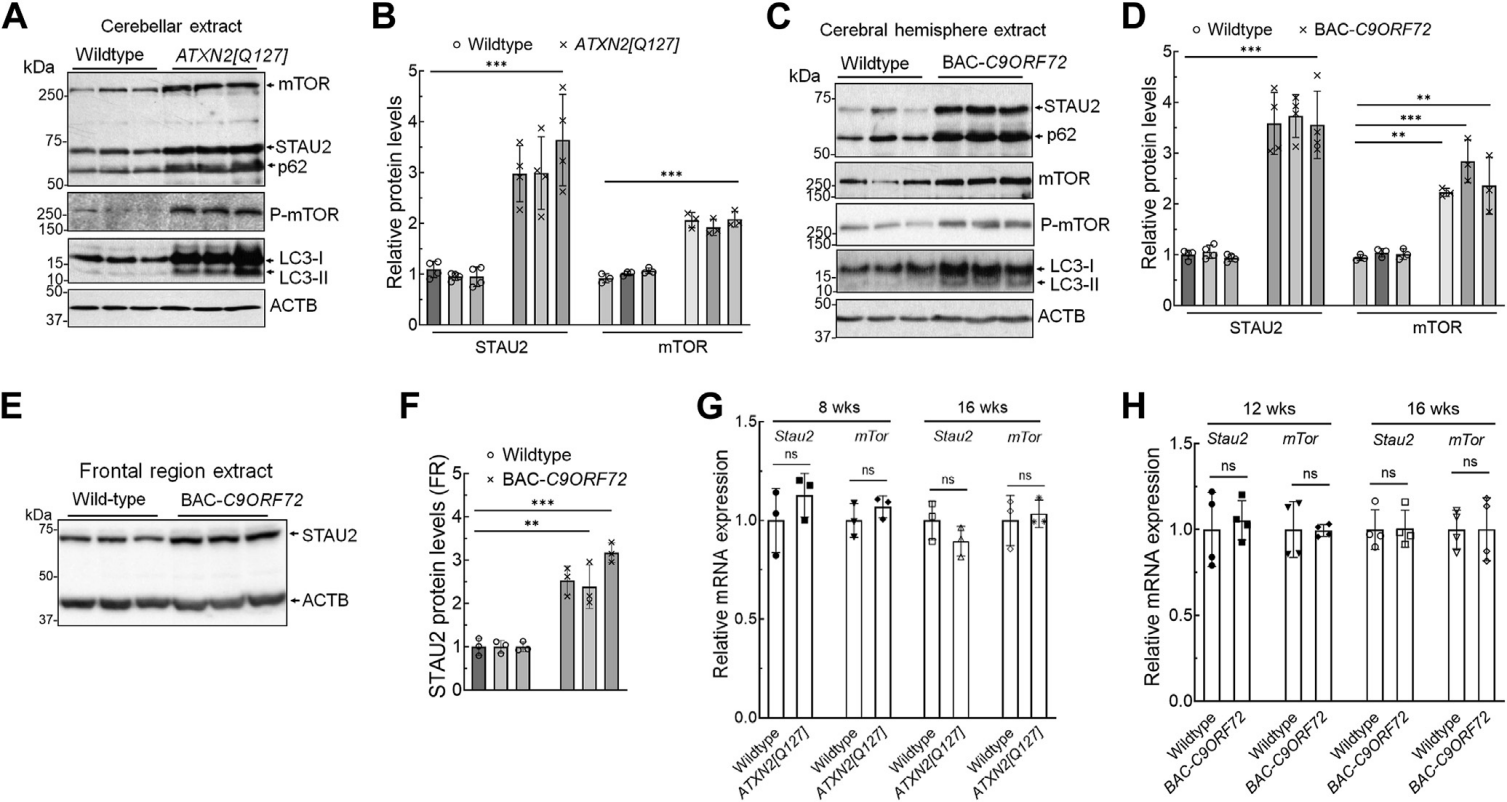
**Central nervous system tissues from mice transgenic for *ATXN2[Q127] or BAC-C9ORF72* have increased STAU2 and mTOR levels.** Western blot analyses of protein extracts from cerebella of *ATXN2[Q127]* mice (16 weeks of age) (*A* and *B*) and cerebral hemisphere (*C* and *D*) of BAC-*C9ORF72* mice (16 weeks of age) show increased STAU2, mTOR, P-mTOR, p62, and LC3-II levels compared with WT controls. *E* and *F*, protein extracts from frontal region of cerebral hemisphere of BAC-*C9ORF72* mice (16 weeks of age) show increased STAU2 levels compared with WT controls on Western blot. Each lane represents an individual mouse, and three animals per group were analyzed. Protein levels were normalized to ACTB, and quantified average fold changes for STAU2 and/or mTOR are shown in *B*, *D*, and *F*. Blots are from three/four technical replicate experiments. *G* and *H*, *Stau2* and *mTor* RNA levels are unaltered in neurodegenerative disease tissues. qRT–PCR analyses of *Stau2* and *mTor* mRNAs from cerebella from *ATXN2[Q127]* mice (8 and 16 weeks of age; three animals per group) (*G*) and cerebral hemisphere from BAC-*C9ORF72* mice (12 and 16 weeks of age, four animals per group) (*H*) compared with WT littermates. Gene expression levels were normalized to *Actb*. Two-way ANOVA followed by Bonferroni tests for multiple comparisons. Data are mean ± SD, ns, *p* > 0.05; *∗∗p* < 0.01; *∗∗∗p* < 0.001. ACTB, β-actin; mTOR, mechanistic target of rapamycin; ns, not significant; P-mTOR, phosphor-mTOR; qRT–PCR, quantitative RT–PCR; STAU2, Staufen2.

We generated and extensively characterized two animal models of SCA2, BAC-*ATXN2[Q72]* and *Pcp2-ATXN2[Q127]* (*ATXN2[Q127]*), with regard to expression of STAU1 and protein markers of autophagy (9, 15, 16). Here, we analyzed protein extracts of cerebella from mice transgenic for *Pcp2-ATXN2[Q127]* (39) at 16 weeks of age (Fig. 2, *A* and *B*) and cerebral hemisphere from mice expressing a human BAC with *C9ORF72* repeat expansion (BAC-*C9ORF72* [C9-500]) (40) also at 16 weeks of age (Fig. 2, *C* and *D*). Western blot analyses revealed increased STAU2, mTOR, P-mTOR, p62, and LC3-II levels in both mouse lines compared with WT littermate controls (Fig. 2, *A*–*D*). Similar to the result for *ATXN2[Q127]* mice, STAU2 overabundance was also observed in protein extracts from the frontal region of the cerebral hemisphere of BAC-*C9ORF72* mice (16 weeks of age) by Western blot analysis (Fig. 2, *E* and *F*). Quantified fold changes for STAU2 and mTOR proteins are shown in Figure 2, *B*, *D* and *F*. However, RNA levels for *Stau2* and *mTor* were unchanged in *ATXN2[Q127]* (Fig. 2*G*) and BAC-*C9ORF72* mice (Fig. 2*H*). We chose to investigate BAC-*C9ORF72* mice of 16 weeks of age, as they first exhibit behavioral motor abnormalities beginning at this age, with a subset demonstrating decreased survival starting at ∼20 weeks (40). We did not observe visible phenotypic changes at 16 weeks or thereafter in keeping with observations by other laboratories that have analyzed this BAC-*C9ORF72* mouse model (41). At a cellular level, however, we detected clear abnormalities when we examined protein markers of spinal motor neurons like choline O-acetyltransferase or neuronal markers like RNA binding fox-1 homolog 3/NEUN by semiquantitative Western blotting. We also observed a clear increase in glial fibrillary acidic protein consistent with glial activation. These observations are consistent with neuronal loss or dysfunction and glial activation at 16 weeks (unpublished observations from our laboratory data). Additional analyses of key autophagic proteins indicated reduced autophagy (Fig. 2, *C* and *D*) and activation of caspase-mediated apoptosis (unpublished observations from our laboratory data).

These results relating to STAU2 overabundance parallel those obtained with STAU1 (9, 15, 16). This also pertains to the replication of altered autophagy function observed in the SCA2 and ALS–FTD cellular and animal models that have overabundant STAU1, as we previously reported (9, 15, 16). In this study, our aim was to elucidate STAU2 functions in regulating the autophagy pathway in the context of STAU2, a paralog of STAU1. Our data in mouse and human tissue, as well as in cellular NDD models, now show STAU2 overabundance accompanied by mTOR activation and its downstream targets: P-mTOR, p62, and LC3-II. STAU2 overabundance is not because of increased transcription, as levels of *STAU2* mRNA were unchanged. It is likely that mutations in NDD-associated genes induce STAU2 overabundance through impairment of autophagy pathways as seen with STAU1 overabundance in NDDs (9, 15, 16). Despite the partially overlapping functions, it may not be surprising that the two members of the Staufen family of proteins exhibit similar changes in cellular and animal models of NDDs. Conversely, a potential reciprocal interaction could have been anticipated, in which an increase in one Staufen protein would have resulted in a decrease in the other. STAU2 functions in RNA–protein SG formation and mRNA localization and transport in brain (22, 24); however, the role of STAU2 in neurodegeneration has received little attention. Direct mutations in STAU2 have not been described, but the several-fold increase in steady-state levels of STAU2 in SCA2 and ALS–FTD-C9ORF72 cellular and animal models, and ALS human tissues suggested a potentially important role in disease.

### Exogenous STAU2 recapitulates mTOR elevation and forms cytoplasmic SG-like aggregates

**Figure 3:**
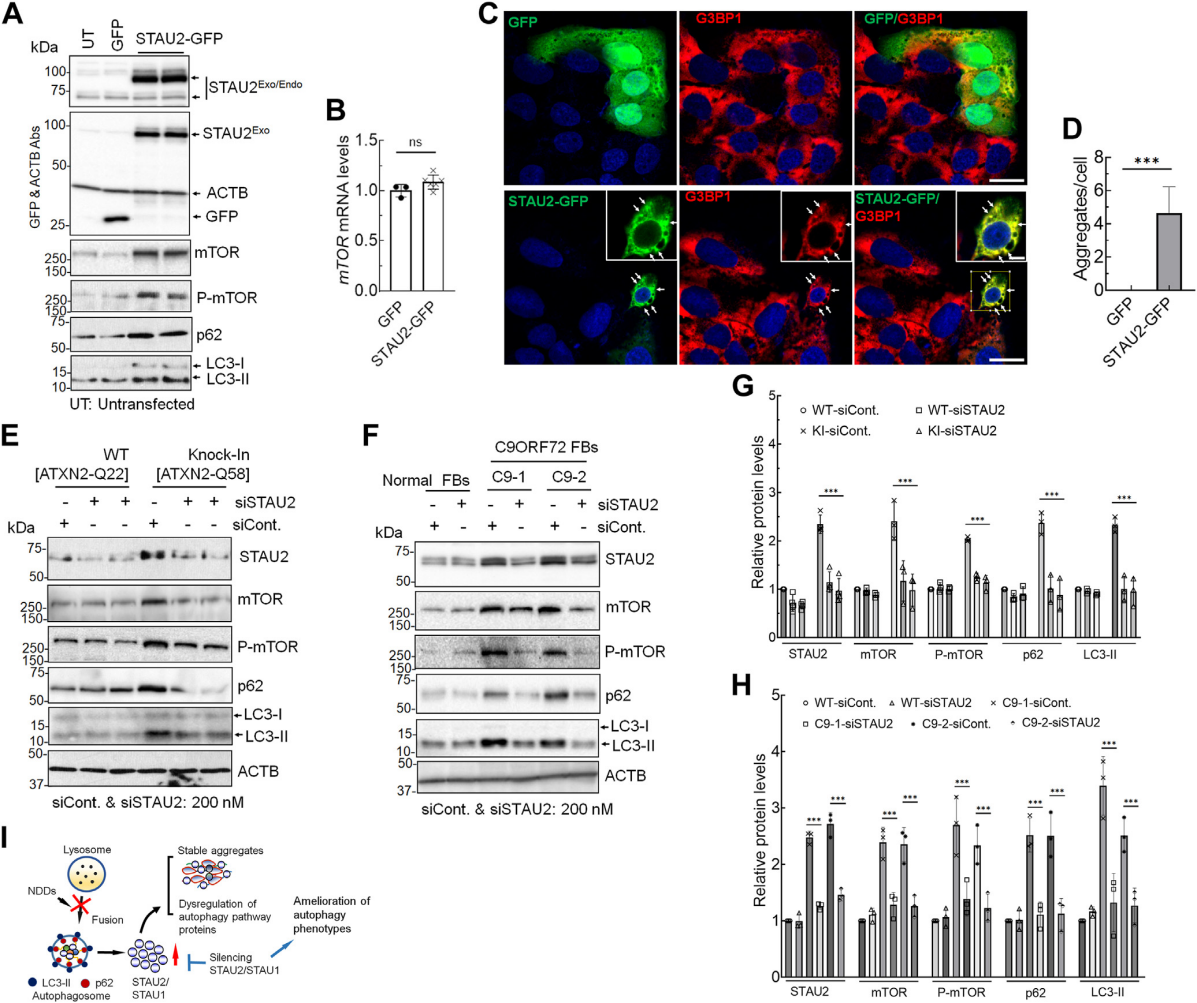
**Overexpression of STAU2 induces mTOR abundance, whereas lowering STAU2 reduces mTOR levels in SCA2 and ALS–FTD cellular models.***A*, HEK293 cells exogenously expressing GFP-tagged STAU2 (STAU2-GFP) or GFP were analyzed 48 h post-transfection by Western blotting and showed increased levels of mTOR, P-mTOR, p62, and LC3-II. *B*, *mTOR* mRNA levels, determined by qRT–PCR, remained unchanged in cells overexpressing STAU2-GFP compared with GFP controls. RNA expression levels were normalized to *ACTB*. Data are mean ± SD, ns = *p* > 0.05, Student's *t* test. *C*, ectopic expression of STAU2 leads to formation of cytoplasmic SG-like aggregates. U2OS cells were transfected with STAU2-GFP or GFP plasmids for 48 h followed by immunostaining with the SG marker protein G3BP1. Cells expressing STAU2-GFP (*lower panel*), but not GFP (*upper panel*), form spontaneous cytoplasmic SG-like aggregates positive for G3BP1 (*merged image*, *yellow*). Scale bar represents zoom in and out 10 and 20 μM, respectively. *D*, quantifications of STAU2 colocalizations with G3BP1 are shown. Fifty GFP-transfected cells (control) of 176 cells and 31 STAU2-GFP-transfected cells of 105 cells were used for analysis. Data are mean ± SD, ∗∗∗*p* < 0.001, Student’s *t* test. *E*–*H*, lowering STAU2 levels by RNAi reduces mTOR activation in SCA2 and ALS–FTD cellular models. HEK293-ATXN2-Q58 KI SCA2 cells (*E*) or ALS–FTD-C9ORF72 FBs (C9-1 and C9-2) (*F*) were transfected with *STAU2* siRNA for 4 days and analyzed by Western blotting. Reducing STAU2 levels in both SCA2 and ALS–FTD cells resulted in decreased levels of mTOR, P-mTOR, p62, and LC3-II compared with cells treated with control siRNA, indicating restored autophagy activity. ACTB was used as a loading control, and quantification of protein levels (*E* and *F*) is shown in *G* and *H*. The blots are from three replicate experiments. Two-way ANOVA followed by Bonferroni tests for multiple comparisons. Data are mean ± SD, ns, *p* > 0.05; ∗∗*p* < 0.01; *∗∗∗p* < 0.001. *I*, model for STAU2 in the pathology of SCA2 and other neurodegenerative diseases. ACTB, β-actin; ALS, amyotrophic lateral sclerosis; FB, fibroblast; FTD, frontotemporal dementia; HEK293, human embryonic kidney 293 cell line; mTOR, mechanistic target of rapamycin; ns, not significant; qRT–PCR, quantitative RT–PCR; SCA2, spinocerebellar ataxia type 2; SG, stress granule; STAU2, Staufen2.

Prior experiments had shown that STAU1 overexpression in normal cells led to cytoplasmic STAU1 aggregates and an increase in mTOR levels and activated caspase in the presence of mutant disease proteins (15, 16, 17). To determine if similar effects could be observed with STAU2, we exogenously expressed GFP-tagged STAU2 (STAU2-GFP) in HEK293 cells. Forty-eight hours post-transfection, Western blot analysis showed increased levels of mTOR, P-mTOR, p62, and LC3-II (Fig. 3*A*), whereas levels of *mTOR* mRNA remained unchanged (Fig. 3*B*), indicating that STAU2-mediated mTOR abundance is associated with post-transcriptional modification. The latter was shown to be the case for STAU1 that binds to the 5′UTR of mTOR, enhancing its translation (16). Pathological protein aggregations are a hallmark of many NDDs, including ALS–FTD, SCA, and Alzheimer’s disease (42). To investigate if STAU2 overabundance was sufficient to induce SG-like structures, we transiently expressed STAU2-GFP in U2OS osteosarcoma cells, frequently used for SG analysis. At 48 h after transfection, STAU2-GFP-expressing cells, but not cells expressing just GFP, exhibited constitutive cytoplasmic aggregates positive for G3BP1, a marker for SGs (Fig. 3, *C* and *D*), indicating that exogenous STAU2 alone was sufficient to drive SG formation in U2OS cells.

SGs/RNA–protein granules are critical for normal RNA metabolism as they regulate translation during cellular stress. RNP granules can regulate mRNA transport and exert local expression control, and SGs are regulated by autophagy. Many protein components of RNP granules are recruited to modulate formation of SGs, including ATXN2, TDP-43, fused in sarcoma (FUS), survival of motor neuron (SMN), fragile X mental retardation protein (FMRP), heterogeneous nuclear RNP A2/B1 (hnRNPA2/B1), Matrin-3 (MATR3), STAU1, and STAU2 (2). Misregulation of these RBPs by mutation or other means contributes to neuronal dysfunctions and is a primary cause of multiple NDDs. Both STAU1 and STAU2 are recruited to cytoplasmic aggregates in brain oligodendrocytes and neurons and modulate SG dynamics (22, 24, 30, 43). Expanded G_4_C_2_ tracts in *C9ORF72* express toxic dipeptide repeat proteins/repeat-associated non-AUG proteins (poly-GA, -GP, -GR, -PR, and -PA) and show predominantly neuronal cytoplasmic aggregates in ALS–FTD patients’ brain frontal cortex or cerebellum (38, 44, 45, 46). Recent studies showed both STAU1 and STAU2 in the dipeptide repeat protein interactome (31). Using transient coexpression, they demonstrated colocalization of poly-GR and STAU1/2 resembling SGs in rat primary neuron cultures. STAU2–poly-GR aggregates were also evident in frontal cortex of ALS–FTD-C9ORF72 patient tissues (31). Our observations of STAU2 overabundance across NDD models, and STAU2-mediated mTOR overabundance, associated with SG production, support the hypothesis that STAU2 has a role in neurodegeneration. It is possible that STAU2 is involved in autophagic feedback regulation on aggregate-prone disease proteins.

### Reduction of STAU2 by RNAi normalizes mTOR levels in SCA2 and ALS–FTD cells

We previously demonstrated that lowering STAU1 protein levels reduced mTOR activation in SCA2 and ALS–FTD cellular and animal models (15, 16). Concomitant with STAU2 overexpression in HEK293 cells and its overabundance in animal models of NDDs, we observed increased mTOR and P-mTOR protein abundance but not increased *mTOR* mRNA (Fig. 3, *A* and *B*). These changes led to increased levels of p62 and LC3-II as a sign of reduced autophagosome–lysosome fusion and impaired autophagy, which are consistent with our previous observations (9, 15, 16). If STAU2 directly contributes to altered autophagy in the presence of mutant genes, its knockdown should normalize mTOR levels and downstream targets. Using an siRNA specifically targeting *STAU2* (32), but not *STAU1*, we transfected SCA2-ATXN2-Q22/Q58 KI cells with *STAU2* siRNA for 4 days and analyzed protein extracts by Western blotting. As patient-derived ALS–FTD-C9ORF72 FBs showed STAU2 overabundance (Fig. 1*D*), we also treated two C9ORF72 FB lines with *STAU2* siRNA for 4 days and analyzed protein extracts. Lowering STAU2 levels in both SCA2 and ALS–FTD cellular models resulted in decreased levels of mTOR, P-mTOR, p62, and LC3-II, restoring autophagic pathway protein homeostasis (Fig. 3, *E* and *F*). Quantification of STAU2 and mTOR levels is shown in Figure 3, *G* and *H*. Thus, reducing STAU2 levels rescued the effect of mutant genes on mTOR levels observed in neurodegenerative states. Overall, our results demonstrate that mutations in NDD-associated genes and consequent elevation of STAU2 level are dually deleterious affecting autophagy function *via* inefficient autophagosome–lysosome fusion, as summarized in Figure 3*I*. Lowering STAU2 expression may represent a potent therapeutic approach for SCA2 and potentially for ALS as well.

The characterization of genes linked to NDDs, and the underlying molecular pathways, has advanced our understanding of the pathogenesis of NDDs. RNA-targeting therapeutics, including RNAi technology (siRNA, shRNA, and artificial miRNA) and antisense oligonucleotides (ASOs), utilize promising strategies for treating NDDs (47). Delivering shRNAs *via* adeno-associated viruses (AAVs) shows promise for treating NDDs. For instance, AAVs carrying shRNAs targeting the human ataxin 1 (ATXN1) transgene delayed the progression of motor and anatomical symptoms in SCA1 mouse models when infused into the deep cerebellar nucleus (48). While siRNA therapy offers high specificity in silencing NDD-linked genes, delivering siRNA effectively across the blood–brain barrier remains a major challenge, limiting its efficacy, causing potential off-target effects, and degradation of siRNAs through endonuclease or exonuclease activities. Developing more efficient delivery systems is essential for its clinical application in NDDs.

### miR-217 expression is downregulated in neurodegeneration

**Figure 4:**
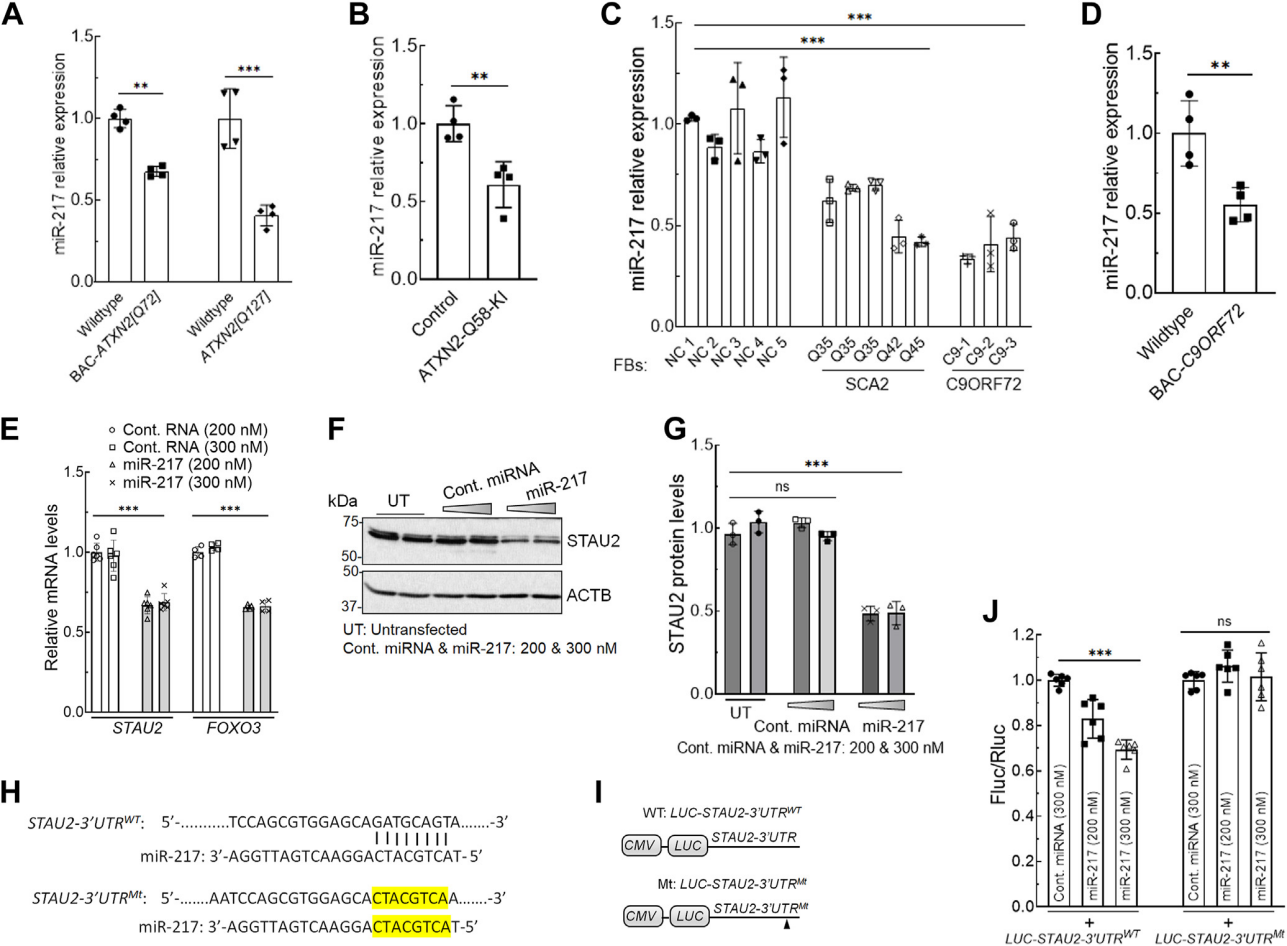
**Evaluation of miR-217 abundance and its regulatory function on its predicted target *STAU2*.***A*–*D*, miR-217 abundances are decreased in SCA2 and ALS–FTD models. qRT–PCR analyses of cerebella from *BAC-ATXN2[Q72]* (16 weeks of age; n = 4) and *ATXN2[Q127]* (14 weeks of age; n = 4) mouse cerebella show decreased miR-217 levels compared with WT littermates (*A*). qRT–PCR analyses of CRISPR–Cas9 edited HEK293-ATXN2-Q58 KI cells *versus* isogenic control cells (*B*), patient-derived fibroblasts (FBs) including three lines from SCA2 patients with the indicated repeat expansions in ATXN2 (Q35, Q42, and Q45 repeats) and three lines from ALS–FTD patients with *C9ORF72* expansions *versus* five normal control (NC) FBs (*C*), and cerebral hemisphere from BAC*-C9ORF72* mice *versus* WT littermates (16 weeks of age; n = 4) (*D*). All show significantly decreased miR-217 levels compared with controls. Gene expression levels were normalized to U6 snRNA. *E*–*G*, miR-217 reduces *STAU2* expression. HEK293 cells were transfected with miR-217 mimic at indicated dosages for 4 days and were analyzed by qRT–PCR and Western blotting. Expression of miR-217 mimic results in reduction of *STAU2* and *FOXO3* (known miR-217 target) transcripts (*E*) and STAU2 protein levels (*F*). Gene expression (*E*)/protein (*F*) levels were normalized to *ACTB*/ACTB, and quantified STAU2 protein levels are shown in *G*. Blots are from three replicate experiments. *H*–*J*, confirmation of miRNA binding to *STAU2-3′UTR*. *H* and *I*, depicted are natural (WT) *LUC-STAU2-3′UTR* (*LUC-STAU2-3′UTR*^*WT*^*)* and predicted miR-217 target site sequence according to TargetScan database. The mutant (Mt) construct (*LUC-STAU2-3′UTR*^*Mt*^*)* with the same *STAU2-3′UTR* sequence except for mutated miR-217-binding site is described in the *Experimental procedures* section. *J*, following transient cotransfection of luciferase constructs, miR-217 mimic, and Renilla luciferase plasmid in HEK293 cells, luciferase activity assays revealed that the miR-217 mimic lowered the expression of luciferase in a dose-dependent manner for WT but not for mutant *STAU2-3′UTR* construct. Two-way ANOVA followed by Bonferroni tests for multiple comparisons (*A*, *C*, *E*, *G*, and *J*) and two-tailed unpaired Student's *t* tests (*B* and *D*). Data are mean ± SD, ns, *p* > 0.05; ∗∗*p* < 0.01; *∗∗∗p* < 0.001. ALS, amyotrophic lateral sclerosis; FTD, frontotemporal dementia; HEK293, human embryonic kidney 293 cell line; ns, not significant; qRT–PCR, quantitative RT–PCR; STAU2, Staufen2; SCA2, spinocerebellar ataxia type 2.

Previously, we analyzed dysregulation of differentially expressed miRNAs (DEmiRs) in cerebellar tissues from BAC-*ATXN2[Q72]* (16 weeks of age) and Pcp2-*ATXN2[Q127]* (14 weeks of age) and confirmed dysregulation in six DEmiRs using quantitative RT–PCR (qRT–PCR) (37). miR-217 is one of six of these DEmiRs that we validated (37). When we analyzed miR-217 using the TargetScan miRNA target prediction database (49) (https://www.targetscan.org/vert_80/), we determined that the 3′-UTR of STAU2 exhibited 8 bp complementarity within the seed region in the 5′-end of miR-217. miR-217 levels were downregulated in both BAC-*ATXN2[Q72]* and Pcp2-*ATXN2[Q127]* mice, determined by qRT–PCR (Fig. 4*A*) (37). We next determined miR-217 levels in HEK293-ATXN2-Q58 KI cells and FBs from patients with SCA2-ATXN2 and ALS–FTD-C9ORF72 repeat expansions using qRT–PCR. In all cases, miR-217 levels were significantly reduced compared with controls (Fig. 4, *B* and *C*). Furthermore, considering the decreased expression of miR-217 levels observed in ALS–FTD-C9ORF72 FBs, we proceeded to determine miR-217 levels in BAC-*C9ORF72* ALS–FTD mice. By qRT–PCR analyses, BAC-*C9ORF72* mouse cerebral hemisphere (16 weeks of age) exhibited significantly decreased miR-217 levels similar to the magnitude of reduction observed in BAC-*ATXN2[Q72]* mice (Fig. 4*D*). Thus, reduced miR-217 abundance is present across different cellular and animal models of human NDDs.

Dysregulated miRNAs induced by NDD-linked genes in the central nervous system have been linked to multiple NDDs, including ALS, Alzheimer’s disease, SCA (SCA1, SCA2, and SCA3), suggesting a role for miRNAs as contributing factors and potential therapeutic targets (37, 50, 51, 52, 53, 54, 55, 56). Our findings demonstrated that both dysregulated miRNAs and their target mRNAs identified in SCA2 mice regulate several neurological disease pathways and are also found to be dysregulated in other NDDs (37, 57). For instance, dysregulation of miR-217 in SCA2 and ALS–FTD-C9ORF72 models (Fig. 4, *A*–*D*) supports this notion. ALS causing mutated protein-driven SGs changes the localization and dynamics of the miRNA-processing enzyme DICER and AGO2 protein, resulting in the dysregulation of mature functional miRNA expression (51). This is consistent with studies demonstrating overall downregulation of miRNAs in ALS patient motor neurons (58, 59). Future work will need to address how common miR-217 changes are across many NDD models and human tissue samples and which NDDs do not show these alterations. It will be particularly instructive to analyze to which degree miR-217 and STAU2 are correlated in different conditions.

### miR-217 represses STAU2 expression

To test whether miR-217 directly regulated STAU2 expression, HEK293 cells were transiently transfected with varying doses of a synthetic miR-217 miRNA mimic for 4 days and analyzed by qRT–PCR and Western blotting. The ectopic expression of miR-217 resulted in a dose-dependent decrease in *STAU2* expression compared with transfection with control miRNA (Fig. 4, *E*–*G*). This decrease was observed at both the transcript and protein levels. In parallel, we also evaluated the effect of miR-217 expression on its known target, forkhead box O3 (FOXO3) (60), as a control. FOXO3 is a transcription factor involved in regulating cell proliferation, apoptosis, metabolism, stress response, and longevity. FOXO3 activation increases the expression of cell cycle inhibitor proteins p21 and p27 as well as apoptosis-inducing genes, *BIM*, *FASL*, *TRAIL*, and *PUMA* (61). As expected, miR-217 expression in HEK293 cells resulted in a dose-dependent decrease in *FOXO3* transcript levels compared with control miRNA (Fig. 4*E*).

### Mapping of miRNA–mRNA binding sites in the 3′UTR of STAU2

miRNAs function as post-transcriptional regulation (degradation or translational inhibition) recognizing specific sequences in the 3′-UTR of target mRNAs. The interaction between miRNA and mRNA greatly relies on the accuracy of the complementarity between the miRNA 5′-end seed region (positions 2–7) and the target mRNA (49, 62, 63). To test if a candidate miRNA directly binds to the regions of STAU2, as predicted by TargetScan database, we performed reporter assays in HEK293 cells. We fused the natural (WT) *STAU2-3′UTR* (*STAU2-3′UTR*^*WT*^) or a mutated *STAU2-3′UTR* (*STAU2-3′UTR*^*Mt*^) sequence lacking the predicted miR-217 binding site to the 3′-end of a luciferase-expressing plasmid (*pCMV-LUC*; described in the *Experimental procedures* section) (Fig. 4, *H* and *I*). Transient cotransfection of *STAU2-3′UTR* LUC reporter constructs, miR-217 mimic, and Renilla luciferase plasmid in HEK293 cells resulted in significantly reduced expression of *LUC-STAU2-3′UTR*^*WT*^, but not of the *LUC-STAU2-3′UTR*^*Mt*^, as compared with control (Fig. 4*J*). This suggests that miR-217 directly binds to the *STAU2* mRNA and regulates its expression.

By the combined analyses of the complementary target sequence and miR-217-predicted targets using TargetScan also identified a number of other targeted genes (*hnRNPA2B1*, *hnRNPA3*, *hnRNPA1*, *RBFOX1/A2BP1*, *FOXO3*, and *SIRT1*), along with STAU2. These encoded proteins are involved in dysfunctional pathways in NDDs (1). *STAU2-3′UTR* represents perfect complementarity to the 5′-end of miR-217 in the seed region with 8 bp (Fig. 4*H*) when compared with the other targets. Given the functions of miRNAs, which have the ability to interact with 3′UTRs to repress their target gene expressions, we focused on the targets that have a high number of complementary base pairings at 5′-ends of miRNAs. Our findings of reduction of endogenous STAU2 and luciferase-STAU2-3′UTR expression upon miR-217 mimic expression strongly support that miR-217 regulates STAU2 expression.

### miR-217 expression lowers STAU2 and mTOR levels in SCA2 and ALS–FTD cellular models

**Figure 5:**
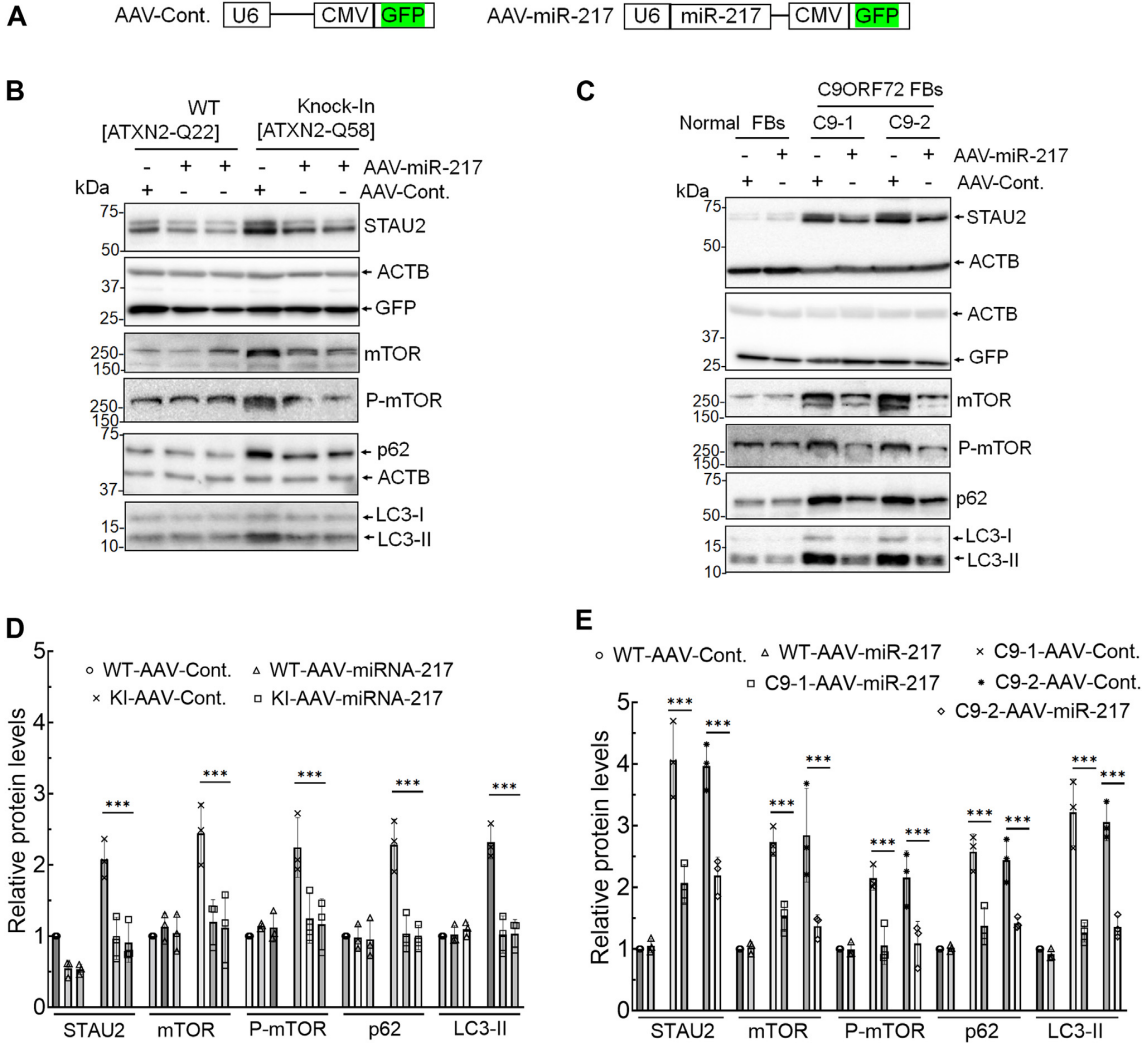
**Targeting STAU2 by miR-217 expression reduces mTOR levels in SCA2 and ALS–FTD cellular models.***A*, schematic depiction of the expression cassettes of the AAV vector plasmids. The AAV vector contains a GFP cassette under control of the CMV promoter and miR-217 sequence under U6 promoter. *B* and *C*, HEK293-ATXN2-Q58 KI cells (*B*) and ALS–FTD-C9ORF72 FBs (C9-1 and C9-2) (*C*) were transduced with control or miR-217 AAV particles for 5 days and analyzed by Western blotting. In both cases, miR-217 expression results in decreased STAU2 levels, as well as decreased mTOR, P-mTOR, p62, and LC3-II levels, indicating restored autophagy activity. ACTB was used as a loading control, and quantification of protein levels (*B* and *C*) is shown in *D* and *E*. The blots are from three replicate experiments. Two-way ANOVA followed by Bonferroni tests for multiple comparisons. Data are mean ± SD, ns, *p* > 0.05; *∗∗∗p* < 0.001. AAV, adeno-associated virus; ACTB, β-actin; ALS, amyotrophic lateral sclerosis; CMV, cytomegalo virus; FB, fibroblast; FTD, frontotemporal dementia; HEK293, human embryonic kidney 293 cell line; mTOR, mechanistic target of rapamycin; ns, not significant; P-mTOR; phospho-mTOR; SCA2, spinocerebellar ataxia type 2; STAU2, Staufen2.

To verify the predicted action of miR-217 on STAU2, we investigated the effects of ectopic miR-217 expression on STAU2 expression in cellular models (Fig. 4, *E*–*G*). As miR-217 negatively regulated STAU2 expression, we generated recombinant AAV with PHP.eB serotype that expressed miR-217 under control of U6 promoter and an independent GFP cassette under control of the CMV (cytomegalovirus) promoter. We observed efficient transduction in all HEK293-ATXN2-Q58 KI cells and C9ORF72 FB lines, with over 80% of cells exhibiting GFP fluorescence. Ectopic expression of miR-217 in HEK293-ATXN2-Q58 KI cells and in FBs from ALS–FTD-C9ORF72 patients resulted in reduced STAU2, mTOR, P-mTOR, and other autophagy protein markers (Fig. 5). This confirms a critical role for miR-217 in autophagy regulation, which is at least in part, mediated *via* STAU2.

As the abundance of both STAU2 and STAU1 associated with abundance and phosphorylation of autophagy markers, the efficacy of the therapeutic knockdown of one may be supported by conservation of partial function by the nontargeted paralog. For STAU1, we understand the direct mechanistic link to autophagy in the control of mTOR translation (16). At the moment, we do not understand if STAU2 directly targets any specific mRNAs controlling autophagy, which will need to be investigated in future work. Our experiments using STAU2 knockdown and ectopic expression, however, are consistent with this potential STAU2 mechanism of action. With regard to therapeutics, ASO efficacy may be in part related to the presence of paralogs, whose expression may mitigate against any loss-of-function effects of the targeted gene. It is also worth noting that *ATXN2* also has a paralog, *ATXN2L*, whose presence might support the efficacy of BIIB105, an *ATXN2* ASO that was tested in a phase I/II clinical trial for ALS (NCT04494256).

Although *STAU1* and *STAU2* do not have sequence similarity as high as *ATXN2* and *ATXN2L* (64), the RBDs in STAU1 and STAU2 are conserved. Despite this, STAU1 and STAU2 mRNA interactomes in HEK293T cells revealed a low percentage of overlap (∼30%) and significant differences in mRNA targets (19). This supports the distinct functions for STAU1 and STAU2 in regulating the localization, transport, and stability of different mRNAs. For instance, in hippocampal neurons, both Staufen proteins form distinct RNA granules that traffic in neurons to distal dendrites *via* microtubules, suggesting unique functions of STAU1 and STAU2 based on their mRNA interactions (22, 28, 65).

Lowering *STAU2* expression directly by siRNA or reducing STAU2 levels with miR-217 expression may be a potent therapeutic approach for SCA2, and it may also be effective for ALS–FTD, where STAU2 and miRNA-217 are dysregulated. Modulation of miRNA expression has been found to be beneficial for NDDs. For instance, overexpression of miR-219 ameliorated tau-induced toxicity in a *Drosophila* model (66). In Parkinson’s disease, expression of miRNAs (miR-7 and miR-153) lowers the expression of synuclein alpha abundance and attenuates dopaminergic neuron degeneration in Parkinson’s disease models (67, 68). In SCA1, expression of miR-19a, miR-101, miR-130a, and miR-144 inhibits *ATXN1* translation *via* cooperative binding to the *ATXN1* 3′-UTR and reverses SCA1-relevant phenotypes (54). Furthermore, through interaction with the 3′-UTR of the ataxin 3 (*ATXN3)* gene, miR-25, miR-9, miR-181a, and miR-494 reduced WT and mutant ATXN3 protein expression, thereby rescuing SCA3 neuronal cell death and mutant ATXN3 protein aggregation (56, 69).

As miR-217 mimic was able to suppress *STAU2* expression in a cell culture model (Fig. 4, *E*–*G*), we demonstrated the restoration of STAU2 levels and mTOR signaling activity upon AAV-mediated miR-217 expression in SCA2 and ALS–FTD-C9ORF72 cellular models (Fig. 5). The negative regulatory effect of miR-217 on *STAU2* expression observed in cellular models raises the question of whether overexpression of miR-217 *in vivo* could mitigate pathogenesis in SCA2 or ALS–FTD models. miRNAs target multiple genes within a pathway, offering significant therapeutic potential. However, they also face significant disadvantages, including potential off-target effects, adverse immune responses, reduced miRNA stability and activity, and difficulties with efficient and specific delivery to target tissues. It is worth noting that broad overexpression of miRNAs with ubiquitous promoters might not be beneficial as miRNAs can regulate a multitude of target genes and cellular processes in unintended organs. However, organ-specific (*e*.*g*., central nervous system) overexpression of miRNAs, such as miRNA-760 and miRNA-17∼92, was found to be beneficial in rescuing neurodegenerative phenotypes in SCA1 and ALS-superoxide dismutase 1 mouse models (70, 71). Future studies are needed to determine if the expression of miR-217 can similarly ameliorate neurodegeneration *in vivo*.

## Conclusions

Our results provide the first description of STAU2 protein levels in models of human NDDs and in human spinal cord tissues from patients with ALS. STAU2 overabundance is not because of increased transcription as levels of *STAU2* mRNA were unchanged. Given the role of NDD-linked genes in abnormal protein homeostasis, we speculate that STAU2 abundance is due to increased stability or impairment of autophagy pathways, as seen with STAU1, a paralog of STAU2 (9, 15, 16). Our data support protein dyshomeostasis as a common underlying feature of NDDs. The increased abundance of STAU2 in NDDs and its association with dysregulation of mTOR pathway predicts that targeting STAU2 may have therapeutic potential. In accordance with this, *ex vivo* STAU2 reduction *via* RNAi showed normalization of proteins in the mTOR pathway in SCA2 and C9ORF72-FTD cellular models. Our data also provide evidence for the negative regulatory effect of miR-217 on *STAU2* expression levels as well as targeting *STAU2* by miR-217 as a means to restore autophagic pathway proteins in a number of NDDs. A more extensive study will need to evaluate whether targeting *STAU2* with ASOs or viral-based therapies can ameliorate neurodegenerative phenotypes *in vitro* and *in vivo*.

## Experimental procedures

### Mouse models

BAC-*ATXN2[Q72]* (BAC-Q72) and *Pcp2-ATXN2[Q127]* (*ATXN2[Q127]*) mice were previously described (34, 39). BAC-*ATXN2[Q72]* transgenic mice are engineered from a 169 kb BAC (RP11–798L5 BAC clone; Empire Genomics) containing the entire 150 kb human *ATXN2* gene (CAG72 repeats) containing all exons, introns, plus 16 kb 5′-flanking and 3 kb 3-’flanking genomic regions (34). *Pcp2-ATXN2[Q127]* transgenic mouse lines were generated using the pGEM construct containing the full human ATXN2 complementary DNA (cDNA) encoding 127 glutamine repeats (Q127) under the control of the *Pcp2* promoter (39). BAC-*ATXN2[Q72]* mice were maintained in 50/50 FVB and then C57B6 background, and *Pcp2-ATXN2[Q127]* mice were maintained in a B6D2F1/J background. BAC-*C9ORF72* mouse (40) commonly referred to as C9-500 (FVB/NJ-Tg(C9orf72)500Lpwr/J) were ordered from Jackson Laboratories (stock #029099). These mice were maintained at Jackson Laboratories in FVB/NJ inbred strain (stock #001800). Upon arrival at our laboratory, the BAC-*C9ORF72* (C9-500) were immediately backcrossed to C57BL/6J (stock #000664), and the experiments were conducted in N3F5 generation. The genotyping of animals was accomplished according to published protocols (34, 39, 40, 72). All mice were bred and maintained under standard conditions in accordance with the National Institutes of Health guidelines and adhered to an approved University of Utah Institutional Animal Care and Use Committee protocol.

### siRNAs and miRNA mimics

The siRNAs used in this study were All Star Negative Control siRNA (Qiagen; catalog no.: 1027280), human siSTAU2: 5′-AGGAAAAGGAGCCGGAUUAdTdT-3′ (32). All siRNA oligonucleotides were synthesized by ThermoFisher. The oligonucleotides were deprotected, and the complementary strands were annealed. The miRNA mimics include mirVana miRNA Mimic, Negative Control #1 (control miRNA) (Ambion/ThermoFisher; catalog no.: 4464058) and mirVana miRNA mimic, hsa-miR-217-5p (assay ID: MC12774; Ambion, Inc/ThermoFisher; catalog no.: 4464066).

### DNA constructs

Human cDNA sequence for *STAU2* (NM_001164380) was derived from the National Center for Biotechnology Information DNA database and used to design primers to PCR-amplify the coding sequences from a cDNA library made from HEK293 cells RNA. The primers set were STAU2-F: 5′-AAAGGATCCAGTTCTCTGTAGTGTTTGCCAATGTTG-3′ and STAU2-R: 5′-AAAGGATCCGACGGCCGAGTTTGATTTCTTGCAGTC-3′. The amplified PCR product was directly cloned into pAAV-CAG-GFP plasmid (gift from Edward Boyden; Addgene plasmid #37825) at BamHI site in frame with GFP, designated as pAAV-CAG-STAU2-GFP. For the natural (WT) Luciferase-*STAU2-3′UTR* (*LUC-STAU2-3′UTR*^*WT*^) reporter assay, we subcloned the luciferase reporter gene from pGL4.10[Luc2] vector (Promega) into pcDNA3.1 (ThermoFisher) at HindIII and XbaI sites, designated as pCMV-LUC. *STAU2-3′UTR* (1003 bp) was PCR amplified from HEK293 cDNA library using following primers: STAU2-3′UTR-XbaI-F: 5′-CCCTCTAGACAGCTCCCAGAACCCGCGGCTGCCACCGC-3′ and STAU2-3′UTR-XbaI-R: 5′-GGGTCTAGATTCACACAGAAACCAACCACATTTTTACTGCATC-3′. The amplified PCR products were cloned downstream of luciferase gene of pCMV-LUC plasmid at XbaI site, designated as pCMV-*LUC-STAU2-3′UTR*^*WT*^. To generate a mutant LUC-*STAU2-3′UTR*^*Mt*^ construct, the following primers were STAU2-3′UTR-*Xba* I-F: 5′-CCCTCTAGACAGCT

CCCAGAACCCGCGGCTGCCACCGC-3′, first STAU2-3′UTR-R: 5′-AACCACATTTTTT

GACGTAGTGCTCCACGCTGGATTCCAAC-3′ and second STAU2-3′UTR-XbaI-R: 5′-AAATCTAGA

TTCACACAGAAACCAACCACATTTTTTGACGTAGTGCTC-3′. The PCR amplifications were carried out with the primers noted above using pCMV-*LUC-STAU2-3′UTR*^*WT*^ as a template, and the amplified PCR products were cloned downstream of luciferase gene of pCMV-LUC plasmid at XbaI site, designated as *pCMV-LUC-STAU2-3′UTR*^*Mt*^. All constructs were verified by sequencing.

### Construction and production of AAV-miR217 vector

The plasmids used to generate AAV include the single-strand AAV expression plasmid, pAAV-U6-sgRNA-CMV-GFP (gift from Hetian Lei; Addgene, plasmid #85451), and pUCmini-iCAP-PHP.eB (gift from Viviana Gradinaru; Addgene, plasmid #103005), and pHelper (Stratagene). For generating AAV-miR-217 expression plasmid, synthesized DNA sequences for miR-217 are miR-217_F: 5′-CGAGTGAGCGCTCCAATCAGTTCCTGATGCAGTACTGTAAAGCCACAGATGGGTACTGCATCAGGAACTGATTGGACGCCTACTATTTTTTA-3′ and miR-217_R: CTAGTAAAAAATAGTAGGCGTCCAATCAGTTCCTATGCAGTACCCATCTGTGGCTTTACAGTACTGCATCAGGAACTGATTGGAGCGCTCACTCGAGCT-3′. After annealing DNA oligonucleotides, miR-217 DNA was cloned downstream of U6 promoter of sgRNA predepleted AAV-U6-CMV-GFP plasmid using SacI and SpeI restriction sites, designated as pAAV-U6-miR-217-CMV-GFP. The plasmid constructs were verified by sequencing. Recombinant AAV particles were generated as published protocol (73) with some modifications. HEK293T cells were cotransfected with three plasmids: either pAAV-U6-CMV-GFP (control) or pAAV-U6-miR-217-CMV-GFP along with pUCmini-iCAP-PHP.eB and pHelper, at a ratio of 1:4:2 (based on micrograms of DNA; ∼30 μg of total DNA per 10 cm dish) using Lipofectamine 2000 transfection reagent (ThermoFisher Scientific) according to the manufacturer’s protocol. About 12 to 18 h post-transfection, media were replaced with fresh culture media and incubated further total of 72 h, and cells were then harvested. Harvested cells were lysed with lysis buffer (25 mM Tris, pH 8.5, 150 mM NaCl) through repeated freezing and thawing cycles using liquid nitrogen and a 37^o^C water bath, followed by centrifugation at 4°C for 30 min at 14,000 rpm. The resultant supernatants containing recombinant AAV particles were filtered with sterile syringe filters, 0.22 μm and stored at −80^o^C until further use.

### Cell line authentication

In order to adhere to the National Institutes of Health guideline on scientific rigor in conducting biomedical research on the use of biological and/or chemical resources (NOT-OD-15-103), we authenticated our cell lines utilizing short tandem repeat analysis on 24 loci (GenePrint 24 system, https://www.promega.com/products/cell-authentication-sample-identification/mixed-sample-analysis/geneprint-24-system/?catNum=B1870).

### Cell culture, transfections, and human tissue specimens

All human subjects gave written consent, and all procedures were approved by the Institutional Review Board at the University of Utah. Two normal controls (non-SCA2 or ALS–FTD) and five SCA2 patient-derived skin FBs were established from local individuals. The following primary human FBs were obtained from the Coriell Cell Repositories (Camden): normal (#ND29510, #ND34769, and #ND38530), two from patients at risk of FTD with *C9ORF72* expansions (#ND42504 and #ND42506), one from a patient with parkinsonism with a *C9ORF72* expansion (#ND40069). Human bone osteosarcoma epithelial cells (U2OS) were obtained from American Type Culture Collection (catalog no.: HTB-96). All FBs, including U2OS and HEK293-ATXN2-Q22/58 KI cells (9, 15, 16) used in this study were maintained in Dulbecco's modified Eagle's medium containing 10% fetal bovine serum and tested for mycoplasma. Spinal cord tissues were received from the Target ALS *Postmortem* Tissue Core (Johns Hopkins School of Medicine, Department of Neurology). These included three non-ALS–FTD control specimens (JHU 96, JHU 101, and JHU 123), four ALS–FTD specimens with *C9ORF72* expansions (JHU 88, JHU 92, JHU 119, and JHU 120), and four specimens from patients with sALS (JHU 102, JHU 111, JHU 113, and JHU 115).

Transfections of siRNAs or plasmid constructs were followed as described (9, 15, 16). For overexpression of recombinant proteins, HEK293 cells were plated on 6-well dishes and incubated overnight. The cells were then transfected with plasmid DNAs and harvested 48 h post-transfection and processed as two aliquots for protein and RNA analyses. For siRNA experiments, cells were transfected with siRNAs using Lipofectamine 2000 transfection reagent (ThermoFisher Scientific) according to the manufacturer’s protocol. Prior standardization experiments showed that maximum silencing was achieved 4 to 5 days post-transfection.

For Luciferase reporter assays, HEK293 cells were cultured in 12-well plates and incubated overnight. Cells were then transfected using Lipofectamine 2000 transfection reagent with the following plasmid constructs and reagents: *pCMV-LUC-STAU2-3′UTR*^*WT*^, *pCMV-LUC-STAU2-3′UTR*^*Mt*^, hsa-*miR-217-5p* (mirVana miRNA mimic), control miRNA, and Renilla luciferase plasmid (pRL-SV40 vector; Promega) according to experimental set-up. Forty-eight hours post-transfection, cells were lysed, and luciferase activity was measured using the Dual-Glo Luciferase Assay System (Promega) on a multimode plate reader (Beckman DT880). Relative luciferase activity was calculated as the ratio of firefly luciferase (Fluc) to Renilla luciferase (Rluc) activity.

### Preparation of protein lysates and Western blotting

Cellular extracts were prepared by a single-step lysis method (9, 15, 16). The harvested cells were suspended in SDS-PAGE sample buffer (Laemmli sample buffer [Bio-Rad, catalog no.: 161-0737]) and then boiled for 5 min. Equal amounts of the extracts were used for Western blot analyses. Mouse protein extracts were prepared by previously published methods (9, 15, 16). Briefly, protein extracts from mouse cerebellar or cerebral hemisphere or frontal region of cerebral hemisphere tissues were prepared by homogenization in an extraction buffer (25 mM Tris–HCl [pH 7.6], 300 mM NaCl, 0.5% Nonidet P-40, 2 mM EDTA, 2 mM MgCl_2_, 0.5 M urea, and protease inhibitors; Sigma; catalog no.: P-8340). The homogenates were subjected to sonication (3 s for one stroke, and level 2; Sonic Dismembrator, Fisher Scientific) followed by centrifugation at 4°C for 20 min at 14,000 rpm. Only the supernatants were used for Western blotting. Protein extracts were resolved by SDS-PAGE and transferred to Hybond P membranes (Amersham Bioscience, Inc). After blocking with 3% skim milk in 0.1% Tween-20/PBS, the membranes were incubated with respective primary antibodies in 3% skim milk in 0.1% Tween-20/PBS for 3 h or overnight at 4°C. After washing in 0.1% Tween-20/PBS, the membranes were incubated with the corresponding secondary antibodies conjugated with horseradish peroxidase (HRP) in 3% skim milk in 0.1% Tween-20/PBS for 2 h at 4°C and washed again. Signals were developed by using the Immobilon Western Chemiluminescent HRP Substrate (EMD Millipore; catalog no.: WBKLSO500) according to the manufacturer’s protocol and detected using a ChemiDoc MP imager (Bio-Rad Laboratories). The band intensities were quantified by Fiji ImageJ (https://imagej.net/Fiji) software analyses after inversion of the images, and proteins were quantitated as a ratio to β-actin (ACTB) or GAPDH. We used Precision Plus Protein Dual Color Standards (Bio-Rad, Inc, catalog no.: 1610374) for SDS-PAGE analyses. For SDS-PAGE and Western blotting analyses, we cast gels in-house to accommodate the proteins of interest being analyzed. Consequently, the migration distances of molecular weight standards on gels may vary depending on the acrylamide gel concentrations used for the analyses.

### Antibodies used for Western blotting

The antibodies used for Western blotting and their dilutions were as follows: mouse anti-ATXN2 antibody (Clone 22/ATXN2) (1:4000 dilution, BD Biosciences, catalog no.: 611378), LC3B Antibody (1:7000 dilution, Novus Biologicals, catalog no.: NB100-2220), SQSTM1/p62 antibody (1:4000 dilution, Cell Signaling, catalog no.: 5114), mTOR antibody (1:4000 dilution, Cell Signaling, catalog no.: 2972), Phospho-mTOR (Ser2448) antibody (1:3000 dilution, Cell Signaling, catalog no.: 2971), GAPDH (14C10) rabbit mAb (1:5000 dilution, Cell Signaling, catalog no.: 2118), GFP (D5.1) rabbit mAb (1:5000 dilution, Cell Signaling, catalog no.: 2956), and monoclonal anti-β-actin-peroxidase antibody (clone AC-15) (1:30,000 dilution, Sigma–Aldrich, catalog no.: A3854). We used each of these antibodies in our previous publications (9, 15, 16, 74). Western blot data using these antibodies for codevelopment of multiple proteins included details in our previous publications (9, 74), highlighting detection specificity. Additional antibody for Western blotting was STAU2 Polyclonal antibody (1:5000 dilution, Proteintech Group, Inc, catalog no.: 15998-1-AP). The secondary antibodies were peroxidase-conjugated AffiniPure goat anti-rabbit IgG (H + L) antibody (1:5000 dilution, Jackson ImmunoResearch Laboratories, catalog no.: 111-035-144) and anti-mouse IgG, HRP-linked antibody (1:5000 dilution, Cell Signaling, catalog no.: 7076).

### RNA and miRNA expression analyses by qRT–PCR

Total RNA was extracted from HEK293-ATXN2-Q58 KI, SCA2, and ALS–FTD FB cells, and mouse cerebella or cerebral hemisphere using miRNeasy Mini Kit (Qiagen, Inc) according to the manufacturer’s protocol. DNAse I-treated RNAs were used to synthesize cDNA using High-Capacity cDNA Reverse Transcription Kit (ThermoFisher; catalog no.: 4368814) or Taqman Advanced miRNA cDNA Synthesis kit (ThermoFisher; catalog no.: A28007). RT–PCR was performed with the Power SYBR Green PCR Master Mix (Applied Biosystems) or with commercial TaqMan assay probes using QuantStudio 12K (Life Technologies, Inc) at University of Utah core facilities as described (9, 15, 16). The forward (F) and reverse (R) primer sequences for Power SYBR Green qRT–PCR are human STAU2-F: 5′-ATCTACGCTTCCCAAACCAG-3′ and human STAU2-R: 5′-GAATGGCTTTGGATCTAATGGC-3′; human ACTB-F: 5′-GAAAATCTGGCACCACACCT-3′ and human ACTB-R: 5′-TAGCACAG CCTGGATAGCAA-3′; human mTOR-F: 5′-CAGAAGGTGGAGGTGTTTGAG-3′ and human mTOR-R: 5′-TGACATGACCGCTAAAGAACG-3′, mouse Stau2-F: 5′-TTGGCTATAAAGCGTCCACC-3′ and mouse Stau2-R: 5′-GACCACTCCATCCTTTGTTTTC-3′; mouse mTor-F: 5′-ATTCAATCCATAGCCCCGTC-3′ and mouse mTor-R: 5′-TGCATCACTCGTTCATCCTG-3′, mouse Actb-F: 5′-CGTCGACAACGGCTCCGGCATG-3′ and mouse Actb-R: 5′-GGGCCTCGTCACCCACATAGGAG-3′. The TaqMan assay kits include human FOXO3 (Assay ID: Hs04195365_s1), human ACTB (Assay ID: Hs01060665_g1), hsa-miR-217-5p (Assay ID: 478773_mir), mmu-miR-217-5p (Assay ID: mmu480999_mir), obtained from ThermoFisher Scientific. For normalization of the qRT–PCR, the miRNA cDNA was synthesized by TaqMan MicroRNA Reverse Transcription Kit (catalog no.: 4366596; ThermoFisher Scientific) and used to measure U6 snRNA levels (Assay ID: 001973).

### Immunofluorescence

U2OS cells expressing STAU2-EGFP were plated on coverslips overnight. Cells were fixed with 4% paraformaldehyde/PBS for 30 min at room temperature, permeabilized/blocked with 0.3% Triton X-100, and 5% goat serum and processed for immunostaining using corresponding primary and fluorescent secondary antibodies. The nuclei were stained with 4′,6-diamidino-2-phenylindole followed by mounting with Fluoromount-G (Southern Biotech, catalog no.: 0100-01). Images were acquired using confocal microscopy (Nikon A1 Confocal microscope) in the University of Utah cell imaging core lab and analyzed by Nikon EZ-C1 or NIS-Elements AR 4.5 software. Primary and fluorescent secondary antibody dilutions were G3BP1 monoclonal antibody (M01J), clone 2F3 (1:1000 dilution, Abnova, catalog no.: H00010146-M01J), goat anti-Mouse IgG (H + L) highly cross-adsorbed secondary antibody, and Alexa Fluor Plus 594 (1:1000 dilution, Thermo Fisher Scientific, catalog no.: A32742). These antibodies were used for immunofluorescence studies in our previous publication (15).

### Statistical analysis

Statistical analyses were performed using GraphPad Prism 9 software (GraphPad Software, Inc). Two-tailed *t*-tests or two-way ANOVA followed by Bonferroni's multiple comparisons test were used to determine significant differences among groups. The levels of significance were indicated as follows: ∗*p* ≤ 0.05, ∗∗*p* ≤ 0.01, ∗∗∗*p* ≤ 0.001, and not significant = *p* > 0.05. Means ± SDs are presented throughout unless otherwise specified.

## Data availability

The data generated and/or analyzed during the study are available from the corresponding author on request.

## Conflict of interest

The authors declare that they have no conflicts of interest with the contents of this article.
